# Onset of sediment transport is a continuous transition driven by fluid shear and granular creep

**DOI:** 10.1038/ncomms7527

**Published:** 2015-03-09

**Authors:** Morgane Houssais, Carlos P. Ortiz, Douglas J. Durian, Douglas J. Jerolmack

**Affiliations:** 1 Department of Earth and Environmental Science, University of Pennsylvania, Philadelphia, Pennysylvania 19104, USA; 2 Department of Physics and Astronomy, University of Pennsylvania, Philadelphia, Pennysylvania 19104, USA

## Abstract

Fluid-sheared granular transport sculpts landscapes and undermines infrastructure, yet predicting the onset of sediment transport remains notoriously unreliable. For almost a century, this onset has been treated as a discontinuous transition at which hydrodynamic forces overcome gravity-loaded grain–grain friction. Using a custom laminar-shear flume to image slow granular dynamics deep into the bed, here we find that the onset is instead a continuous transition from creeping to granular flow. This transition occurs inside the dense granular bed at a critical viscous number, similar to granular flows and colloidal suspensions and inconsistent with hydrodynamic frameworks. We propose a new phase diagram for sediment transport, where ‘bed load’ is a dense granular flow bounded by creep below and suspension above. Creep is characteristic of disordered solids and reminiscent of soil diffusion on hillslopes. Results provide new predictions for the onset and dynamics of sediment transport that challenge existing models.

## Abstract (web summary)

The onset of sediment transport is commonly believed to be an abrupt process, which influences our understanding of river erosion and landscape dynamics. Houssais *et al.* show instead a continuous transition from a creeping to a granular-flow regime by monitoring internal motion in a sediment bed.

## Graphical abstract

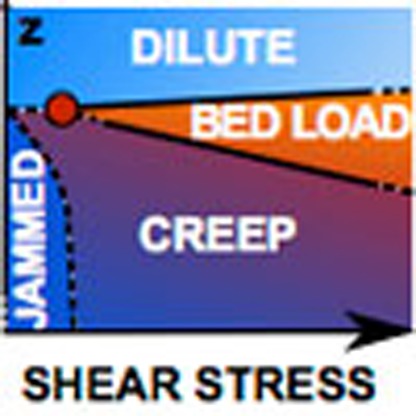

The Earth’s surface is a fluid-sediment interface that evolves through the transport of granular material driven by an applied fluid boundary-shear stress, *τ*; it is often reported as the dimensionless Shields number, 
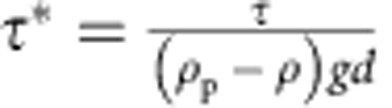
 where *ρ*_p_, *ρ*, *g* and *d* are particle density, fluid density, gravity and particle diameter, respectively. The onset of sediment transport is classically associated with a critical Shields number, 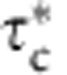, that is required to overcome grain friction^1^,^2^,^3^. For a range of values 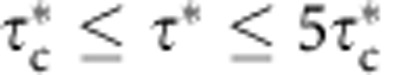, transport occurs as bed load, which is typically viewed as a thin surface layer of moving grains in frequent contact with—and supported by—an underlying granular bed that is totally static^4^,^5^,^6^. For sufficiently large stresses 
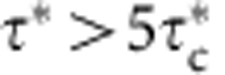
, particles are entrained into the interior of the flow to form a dilute suspension above the bed-load layer^4^.

While models for suspended-sediment transport in both turbulent and laminar flows are reasonably accurate, bed load prediction remains a challenge. Bed-load transport equations typically employ a momentum balance between the near-bed fluid and mobile surface grains, resulting in a nonlinear relation between mass flux and τ* (refs 4, 5, 6). For 
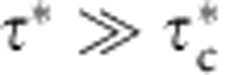
 these equations produce fair agreement with data; however, they break down on approach to 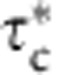 where bed-load flux becomes highly intermittent^7^,^8^,^9^. Unfortunately, near-critical transport predominates in gravel rivers, which erode their banks until the fluid stress on the channel margins is in the vicinity of the threshold of motion^10^. The limiting factor for predicting near-critical transport appears to be determining the threshold of motion itself.

Many studies have emphasized the confounding role of large-scale fluctuations in τ* as a result of turbulence^4^,^8^,^11^,^12^. Recently, however, researchers have begun to recognize the importance of particle–particle interactions in determining both the rheology of^13^,^14^,^15^,^16^ and river patterns resulting from^17^ bed-load transport. Sheared granular materials are known to undergo a transition from a dense liquid-like state to a slow creeping state, as the shear stress crosses the material yield stress^18^,^19^. In such a transition, the timescale of grain dynamics diverges on approach to the critical point^20^. Given that dry-granular flows, colloidal suspensions and even molecular glasses exhibit similar dynamics^18^,^19^,^21^, it seems reasonable to expect that bed-load transport should exhibit the same kind of transition. If it does, it might explain much of the difficulty in determining the onset of sediment transport.

We test the ideas above by examining the dynamics of fluid-driven sediment transport under a laminar shear flow, over a wide range of time scales. Results show that the onset of sediment transport is a continuous transition—from a creeping state to a granular flow—presenting a new view that contradicts the widely accepted static threshold concept. This transition is associated with a critical viscous number. Surprisingly, particle concentration does not vary significantly across the transition. We propose a new phase diagram that unites fluid-driven sediment transport with a broader class of granular systems, while presenting challenges to current models for both.

## Results

### Experimental set-up

**Figure 1:**
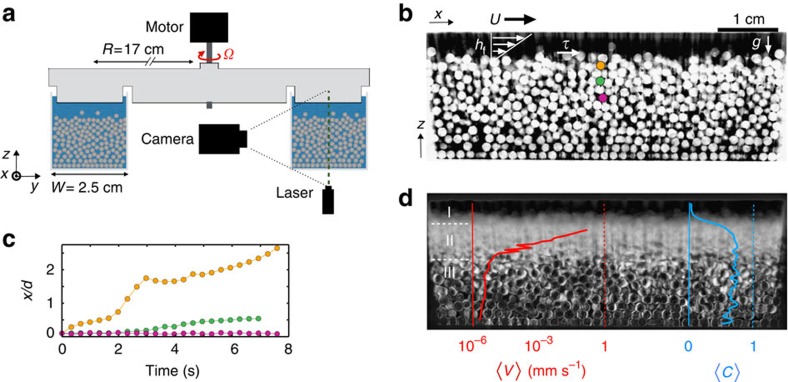
Experimental set-up and definition of variables. (**a**) Cross-section sketch of the experimental set-up. (**b**) Example processed greyscale image of light absorption in the interior of the bed (see details in Supplementary Information); fluid and particles appear as black and white, respectively. Also shown is the fluid boundary stress, which is computed as *τ*= *ηU*/*h*_f_, with *U* the top-plate speed, and *h*_*f*_ the flow depth. Coloured particles correspond to tracks in (**c**), showing horizontal position *x* as a function of time *t*; experiment driven at τ*=0.45. (**d**) Particle activity; image shows pixel-wise s.d. over 10-h run time for the same experiment. Blue and red curves show corresponding long-term-averaged particle concentration ‹*C*› and velocity ‹*V*›, respectively. Three particle regimes discussed in text are noted I, II, III.

To probe the granular dynamics associated with near-threshold bed-load transport, we performed six distinct experiments in an idealized laboratory river consisting of a closed annular flume (Fig. 1). Channel walls are smooth to allow slip between particles and the boundary to approximate an infinitely deep and wide channel. We immerse a granular bed of acrylic spherical particles of diameter *d*=1.5 mm and density *ρ*_p_=1.19 g ml^−1^ in a fluid of viscosity *η*=72.2 mPa s and density *ρ*=1.05 g ml^−1^. The system of width *W*=17 *d* is sheared by rotating the top of the flume at a constant rate Ω, which varied from 0.7 to 4 r.p.m. in our experiments (see Fig. 1a), corresponding to 0.04≤τ*≤0.45 (see details of *τ* measurement in Methods). The low fluid Reynolds number (Re≤3) for all experiments ensures that both turbulence and secondary flows are suppressed^5^ (see Methods), and allows us to isolate the slow dynamics of particles under conditions simulating an infinitely long, straight channel. Although turbulence is an important component of sediment transport, experiments and theory show that laminar bed load is similar to its turbulent counterpart in many respects^5^,^6^,^13^. To visualize granular dynamics, we index-match the particles and the fluid, and record laser-excited fluorescence of a dye dispersed in the fluid^15^,^22^; this allows us to image a vertical profile of grains in the centre of the channel (Fig. 1b; see Methods). By acquiring images at a variety of frame rates for durations up to several days, we are able to resolve particle velocities over seven orders of magnitude (see Supplementary Movies 1–3). Trajectories of individual grains show that transport velocity varies widely, both in time and with depth below the surface (Fig. 1c). By appropriately averaging, however, we are able to recover a mean-field description of the vertical profiles of particle streamwise velocity ‹*V*› and concentration ‹*C*› (Fig. 1d; see also Supplementary Fig. 1). Three different regimes are apparent, moving from top to bottom: (I) an upper layer where ‹*C*› approaches zero and fluid forces dominate; (II) a middle layer where ‹*C*› abruptly saturates to a constant value *C*_sat_ while ‹*V*› continuously decreases exponentially; and (III) a lower layer where ‹*C*› remains constant and ‹*V*› decreases more slowly. It is intriguing that the transition from (II) to (III) is associated with a kink in the particle velocity profile, but no significant change in concentration; we examine these points in detail below.

### Depth-integrated flux

**Figure 2:**
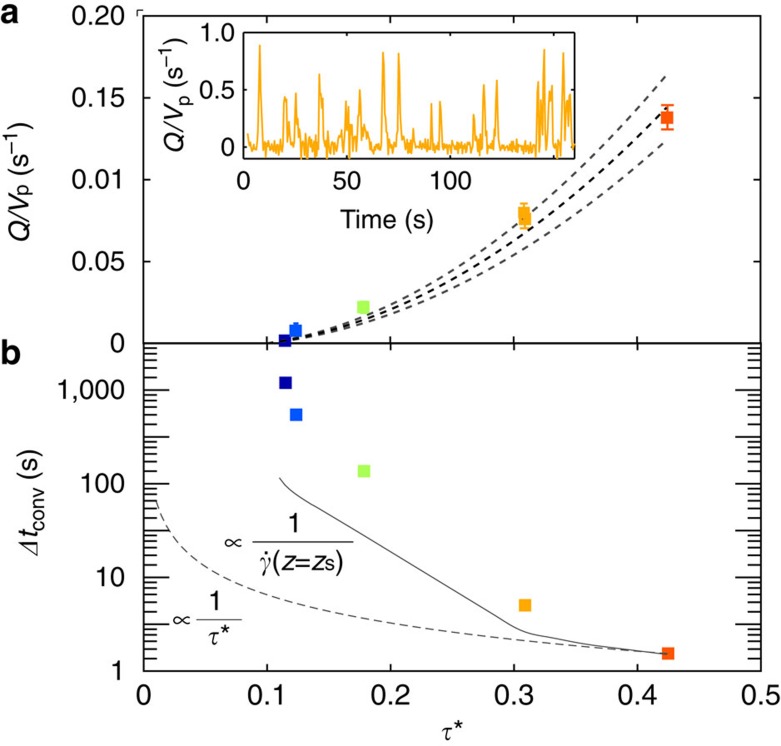
Time-averaged sediment flux. (**a**) Depth-integrated sediment flux *Q* normalized by particle volume *V*_P_, measured mid-channel at a given *x* position (see Methods), as a function of Shields number, indicated by colour; colours apply to all subsequent data. Dotted lines are mean and 90% confidence interval of a previously proposed laminar bed-load relation 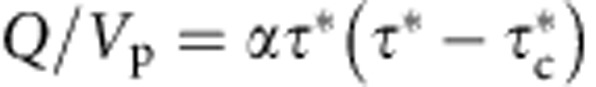, where 
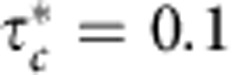
 was chosen based on similar experiments 5 and the fitted value *α*=0.91±0.13 s^−1^. Inset: representative time series of sediment flux, normalized by subtracting its mean value over time ‹*Q*› and dividing by its s.e. *σ*_‹*Q*›_. (**b**) Timescale for flux measurements to converge Δ*t*_conv_ as a function of the Shields number τ*, defined as the minimum time for the relative error *σ*_‹*Q*›_/‹*Q*› to go below a threshold of 60% (see Methods). Uncertainties for both τ* and Δ*t*_conv_ are similar to the size of the markers. The dotted line represents the relation Δ*t*_conv_=1/τ* expected for a fluid strain-rate timescale. The solid curve represents the strain-rate timescale for surface particles, 
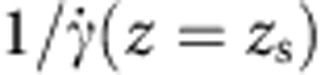
, determined from a spline fit to the data.

We first verify that our experimental results are consistent with expectations of sediment transport derived from previous studies. Even with a laminar flow and uniform spherical particles, the time series of depth-integrated sediment flux is highly intermittent (Fig. 2a); it is qualitatively similar to experiments involving turbulent flows and non-spherical particles^6^,^7^,^8^,^14^. The origins of this erratic stick-slip dynamics must lie in particle–particle interactions, rather than the fluid. In contrast, the average flux—when appropriately computed over a convergence time Δ*t*_conv_—follows the laminar bed-load transport law found earlier^5^,^23^ for a similar experimental set-up (Fig. 2a) and indicates a similar critical Shields number, 
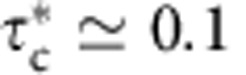
.

A notable result is that Δ*t*_conv_ rapidly diverges as τ* approaches the inferred 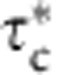. The timescale Δ*t*_conv_ for each experiment is comparable to, but longer than, the strain-rate timescale of surface grains, and is much longer than the fluid strain-rate timescale (Fig. 2b). The diverging timescale is associated with the slowing down, and increasing variability, of particle dynamics on approach to critical; it is unrelated to hydrodynamics. As a consequence, the closer the system is to critical, the longer it is necessary to record particle displacements to compute a flux. A hint of this behaviour can be seen in recent turbulent bed-load experiments^8^. These results suggest that determination of the threshold of motion depends on the resolution of the measurement, and that particle motion may not cease at a clearly defined 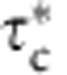. We propose that sediment transport undergoes a continuous slowing down—rather than a discontinuous stop—on approach to its critical point (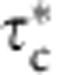), similar to granular and colloidal systems^18^,^20^.

### Long-time ensemble-averaged dynamics

**Figure 3:**
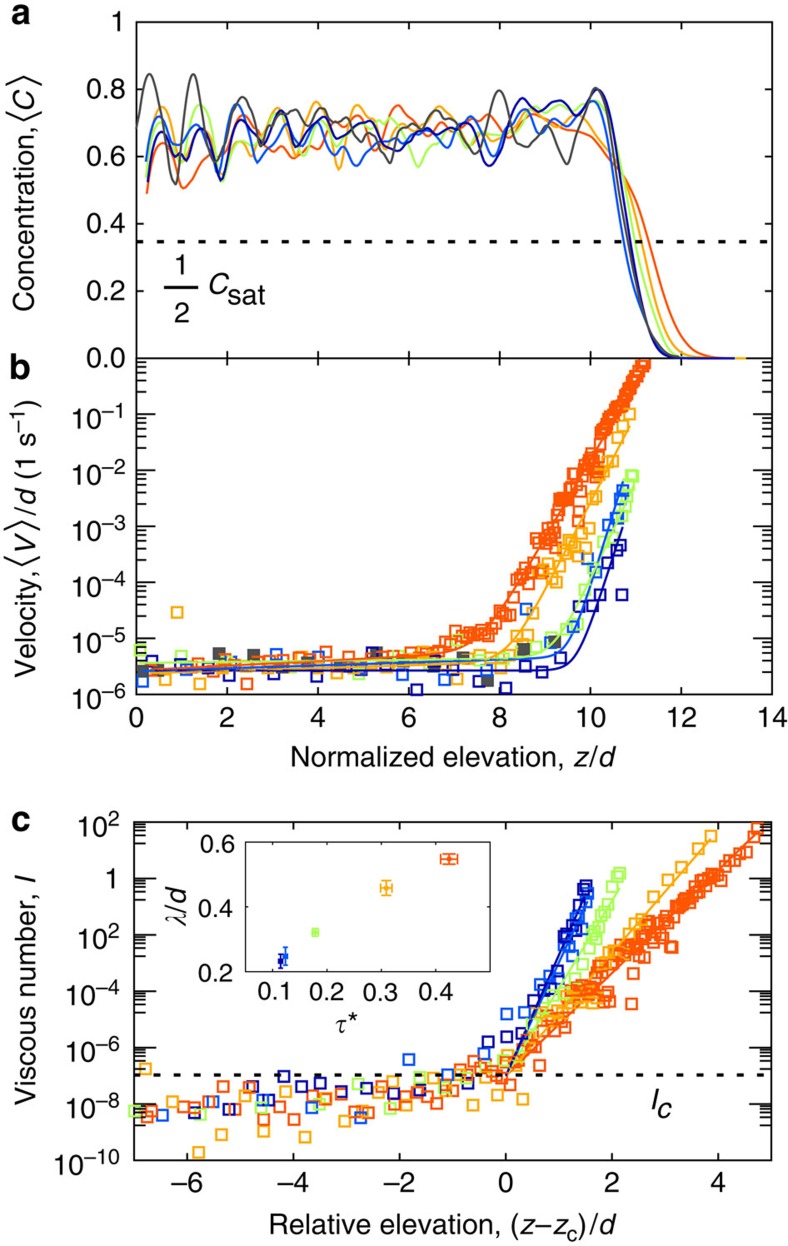
Long-time averaged vertical profiles. Profiles for different values of Shields stress τ*; colours correspond to Fig. 2, *z* is elevation and *d* is grain diameter. In panels (**a**) and (**b**), an additional sub-critical data set at τ*=0.04 is coloured dark grey. Bottom of channel corresponds to *z*/*d*=0. (**a**) Particle concentration ‹*C*›, showing reference value for determining the surface elevation *z*_s_. (**b**) Streamwise particle velocity ‹*V*›. Note the kink apparent in each profile at similar values of ‹*V*›. Except for the sub-critical experiment (τ*=0.04)—which lacks a well-developed regime (II)—profiles are well-fitted by a double-exponential function, which allows characterization of the critical depth *z*_c_ associated with the kink (see Methods). (**c**) Profiles of viscous number *I* (eq. 1), where elevation has been shifted relative to *z*_c_. Exponential fits (eq. 2) to dense-granular flow regime (II) are shown, and were used to compute the lengthscale *λ* (inset). Note profiles converge around the value *I*_c_~10^−7^ shown by dashed line (see Methods), which we propose marks the transition from bed load to creep. Inset: normalized lengthscale *λ*/*d* for the dense-granular flow regime (II) grows in proportion to τ*.

**Figure 4:**
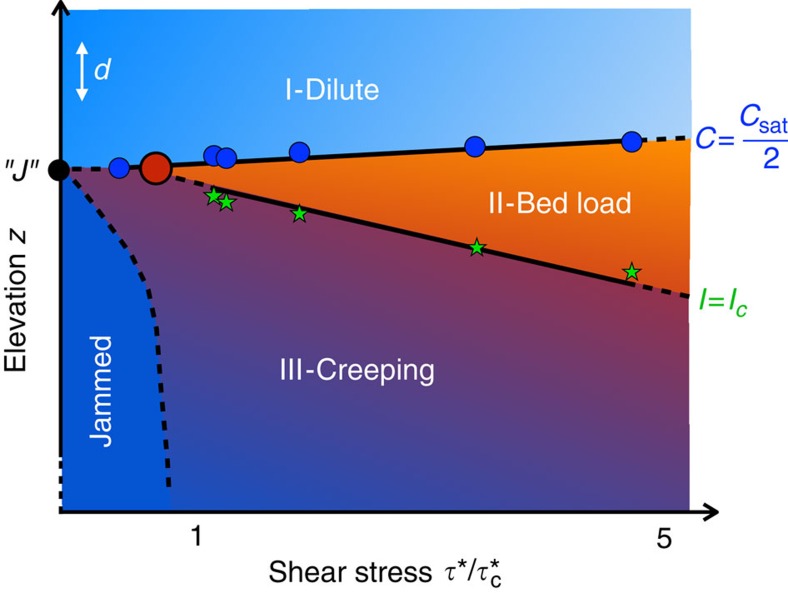
Proposed phase diagram of fluid-sheared granular transport. The fluid shear stress axis is arbitrarily normalized by the value of 
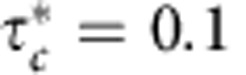
 inferred from particle flux data; *d* indicates grain diameter. Blue circles and green stars are measurements of particle concentration ‹*C*›=*C*_sat_/2 (or *z*=*z*_s_) and viscous number *I*=*I*_c_ (or *z*=*z*_c_), respectively. Data indicate that the bed-load layer thickness 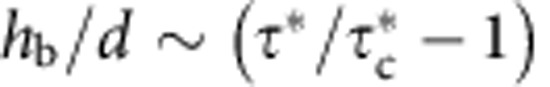 for 
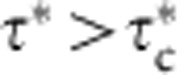
. Regime (I) corresponds to dilute transport in suspension, regime (II) to bed-load transport associated with dense-granular flow dynamics and regime (III) to slow creeping. The red dot marks the bifurcation of regimes (II) and (III) that occurs when the onset of bed-load transport intersects the surface, that is, *z*_s_= *z*_c_ and *h*_b_=0. This point of vanishing bed load provides another estimate for 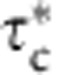, that is somewhat <0.1. The critical inertial number *I*_c_ separates regimes (II) and (III) when *h*_b_>0 and 
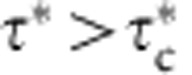
, but is not defined for 
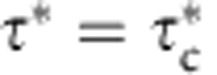
 when *h*_b_=0. Sub-threshold creep occurs for stresses 
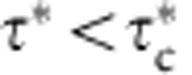
, as indicated by the point to the left of the red dot (no reliable *z*_c_ can be reported for this point since bed load was negligible; point was shifted vertically to account for difference in initial bed thickness compared to other experiments). There must be a sufficiently low stress for which all motion ceases and the granular bed is jammed (point ‘*J*’); its location is currently unknown, so it is conservatively placed at zero stress. All hypothetical regions are denoted by dashed lines; solid lines are delineated from our data but are meant only to guide the eye.

We return to the three particle regimes introduced earlier. Particle concentration profiles (see Methods) all exhibit an abrupt increase with decreasing *z* to achieve a similar saturated value *C*_sat_ inside the bed (Fig. 3a). We define the bed surface elevation *z*_s_ as 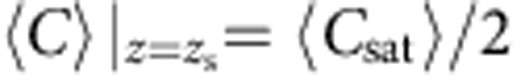 (see Methods). We interpret the change in ‹*C*› from (I) to (II) as a transition from a dilute regime driven by the fluid and gravitational forces, to a dense-granular flow^24^. Particle velocity profiles have two distinct features (Fig. 3b): an exponential decay below the surface corresponding to regime (II), and a transition to a second, weaker exponential-like decay (regime III) that occurs at similar values of ‹*V*› regardless of τ*. (An important exception is the lowest, sub-critical experiment associated with τ*=0.04, for which only regime (III) is well developed). We define the depth of the transition, *z*_c_, as the location of the kink in the profile (Fig. 3b). The thickness of regime (II) may then be characterized as *h*_b_=*z*_s_−*z*_c_, which is zero for subcritical stress and grows with increasing τ* above critical (Fig. 4). To investigate the granular dynamics of regimes (II) and (III), we compute profiles of the viscous number *I*:


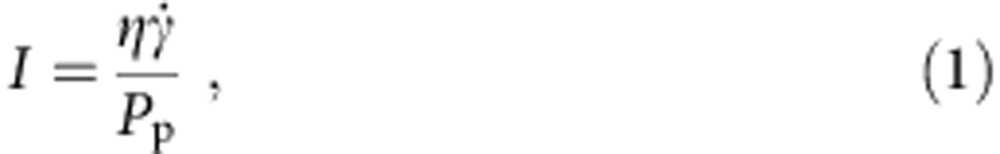


as a function of depth relative to the critical value *z*−*z*_c_, where 
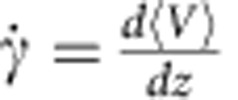
 is the shear rate and *P*_p_ is the confining pressure (see Methods). The viscous number represents the importance of the timescale for confined particles to rearrange in the fluid 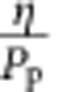 versus the timescale of the strain 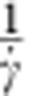, and has recently been proposed as a unifying parameter for characterizing the frictional rheology of dense suspensions and granular flows^25^,^26^. On the basis of comparison with other granular systems^24^,^25^,^26^, we posit that the bed develops a dense-granular flow regime in the elevation range between *z*_s_ and *z*_c_ (regime II), where


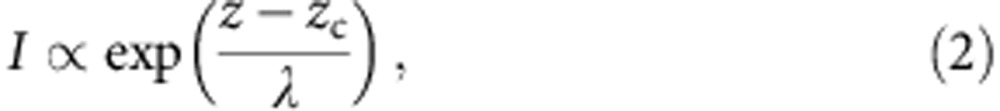


and *λ* is a characteristic decay lengthscale determined from fitting equation 2 to our data (Fig. 3c).

For all values of τ* our experiments exhibit an approximately constant viscous number 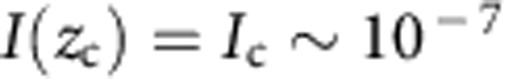 at *z*=*z*_c_, close to values found in recent experiments of sheared-granular systems^19^,^26^. The lengthscale *λ* increases with τ* (Fig. 3c) in the same manner as *h*_b_; their ratio is constant, *h*_b_/*λ*= 3±1. We identify the dense-granular flow regime (II) as the bed-load layer. Recent experiments of bed-load transport, in turbulent flows and with non-spherical particles, have shown qualitatively similar patterns in terms of the particle velocity profile^3^,^14^ and growth of the bed-load layer^14^. The lengthscale *h*_b_ can then be interpreted to represent the thickness of the bed-load layer, while *λ* is a constant fraction of that.

The transition from regime (II) to regime (III) represents the most important finding of our experiments. The decay in viscous number dramatically slows down, and does not go to zero, across the transition. The constant value for *I*_c_ despite variations in τ*, and its coincidence with the minimum values found in other granular flows^19^ and dense suspensions^26^, strongly suggests the presence of a continuous granular-flow transition. At depths below *z*_c_, the viscous number profiles are similar for all experiments (Fig. 3c), indicating similar dynamics. The lower regime (III) consists of exceedingly slow particle motion associated with structural rearrangements of the bed. We identify this as a creeping regime, similar to sub-threshold motion observed in disordered solids^18^,^27^. Interestingly, creeping is commonly associated with the slow, gravity-driven motion of soil that occurs on hillslopes in the absence of fluid-driven transport^28^,^29^. We hypothesize that creeping occurs generally in fluid-driven sediment transport—both below the bed-load layer when it is active and for sub-threshold shear stresses where no bed-load transport occurs. The latter point is supported by our sub-threshold experiment (τ*=0.04), which exhibits creep in the absence of bed load. Creep has likely not been reported previously because sediment transport experiments did not observe particle motion for sufficiently long times. It is also possible that straight flumes suppress creep due to the confining downstream wall that is required to retain grains in the channel; annular flumes do not have this limitation. Although creeping may be influenced by particle shape such that natural river sediments behave differently from our experimental spheres, dry granular flow experiments demonstrate that creeping occurs even for non-spherical particles including natural sand^30^.

## Discussion

In light of these results, we propose a new phase diagram for fluid-sheared granular transport (Fig. 4). A critical Shields number 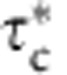 still exists; however, it does not represent a discontinuous threshold of motion; rather, it signals a bifurcation in the transport dynamics between creeping and bed load. Data support a relation for the bed-load layer 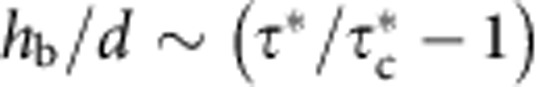 for 
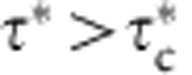
 (Fig. 4), such that the bed-load layer vanishes at a finite Shields number. Owing to subcritical creep, transport does not totally vanish. The bed-load layer is defined at its bottom boundary *z*_c_ by a critical inertial number *I*_c_ associated with the transition to quasi-universal creeping dynamics (III). It is defined at its top boundary *z*_s_ by a particle concentration ‹*C*›=‹*C*_sat_›/2, above which sediment is transported in a dilute regime (I) associated with suspension.

The existence of creeping indicates that the fluid stress applied at the surface is accommodated by motion deep into the bed; this mechanism for attenuation of fluid momentum is unaccounted for in existing models. In particular, state-of-the-art sediment transport models presume a transition to no motion at a critical particle concentration, below which a fixed static friction is applied^12^,^13^,^15^,^31^. This is incompatible with our observed transition to slow creeping at a critical viscous number. The presence of creeping below the bed-load layer is also important for river-scale dynamics. Although its contribution to overall mass flux is negligible for flows in excess of 
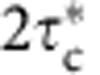
, the creep fraction rapidly approaches one as the stress decreases towards 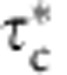 (Supplementary Fig. 8). Moreover, creep could help to explain puzzling laboratory and field observations of apparent temporal changes in the threshold of motion^5^,^7^. Creeping drives compaction of a granular bed, which increases the effective friction and hence the critical stress^26^. Bed load appears to drive dilation, as evidenced by the increase in *z*_s_ with increasing τ* (Fig. 4), which may decrease friction and the critical stress. Furthermore, the diverging timescale associated with the transition to creep (Fig. 2) is a likely contributor to the large scatter observed in measurements of near-threshold bed-load transport^9^, which do not use a dynamically informed averaging timescale.

Finally, long-term models of river evolution typically require specification of an ‘active layer’, which is considered to be the depth over which the sediment bed is mixed by bed-load transport, and granular sorting can occur^4^,^32^. Often this layer is defined subjectively or heuristically. Our new characterization of the bed-load regime provides a mechanistic definition for the active layer that may be directly tested with field data. We are cautiously optimistic that our results are not restricted to the idealized experimental set-up, as our observations of the bed-load layer are consistent with the few reported results from more ‘realistic’ experiments^3^,^14^.

Our results indicate that surface sediment motion is influenced not only by fluid shear from above, but by granular creep from below. Spontaneous and intermittent transport of surface particles occurs even in the absence of turbulence, in part as a consequence of subsurface creeping. These observations stress the importance of modelling granular rheology in the treatment of sediment transport, a perspective that is new but is gaining ground^13^,^14^,^17^. These results also suggest that the phenomenology of granular phase transitions^18^ may be extended to geophysical sediment transport, providing a complementary approach to the prevailing hydrodynamic view.

## Methods

### Experimental set-up and protocol

To obtain clear 2D images of our 3D experimental system, we index-match PMMA particles (*d*=1.5 mm, Engineering Laboratories) with a mixture of viscous oils (85% of PM550 and 15% of PM556 from Dow Corning), and excite a dye (Exciton, pyrromethene 597) dispersed in the oil with a green laser sheet (517 nm, 50 mW) of thickness ≃*d*/10. The experiment is conducted on a vibration-damping optical table, while a damping coupler is used to connect the driving motor to the flume. All computer code used in the methods described below is available for download at http://dx.doi.org/10.6084/m9.figshare.1269323.

The granular bed for each experiment is prepared with the same protocol: for 5 min the flume top is rotated at 30 r.p.m., applying a total shear stress strong enough to suspend all particles. The rotation then slowly returns to zero, and the particles settle for 5 min, building a random packed layer of ~11 *d*. The only exception is the subcritical experiment (τ*=0.04), which was ~13 *d* thick. Then, a constant rotation Ω drives the system during the entire experiment. The duration of the experiment is not fixed; each lasts long enough (10 h to several days) that all particles present in the recorded frames exhibit some significant displacement during the run. The bed exhibits a significant compaction phase at the beginning of the experiment, which involves a slight decrease over time of the bed surface elevation. Thereafter the system reaches a saturated regime, where the overall bed thickness change is <10%. Data are acquired in this steady-state regime, from which we compute the long-time-averaged parameters ‹*C*(*z*)› and ‹*V*(*z*)›.

We compute the fluid-flow depth *h*_*f*_= *H*−*z*_s_, where *H* is the total depth of the flume and the elevation of the surface *z*_*s*_ is determined as shown below. We compute the fluid-flow velocity at the top plate in the channel centre as *U*=Ω·2*πR*, where *R*=17 cm is the radial distance to the channel centre. The fluid boundary-shear stress is then calculated as *τ*= *ηU*/*h*_f_. To compute the confinement pressure profile *P*_p_(*z*), we define a hydrostatic pressure based on the ensemble-averaged particle concentration profile ‹*C*›(*z*): 
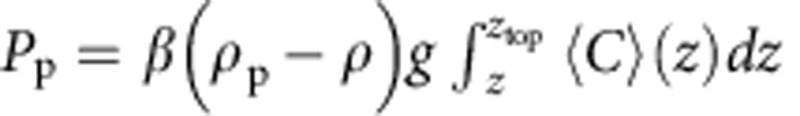
, where *z*_top_ is the elevation of the rotating top plate and *β* is a normalization coefficient equal to 0.836, chosen to scale our 2D concentration ‹*C*_sat_›=0.7 to a 3D packing fraction of 0.585, similar to recent experiments in a comparable system^15^.

For our definition of τ*, and throughout our analysis, we assume the fluid flow is laminar and unidirectional in the azimuthal direction of the annular flume. The laminar assumption is justified because the Reynolds number associated with the fluid channel above the bed is small. We estimate this Reynolds number as Re=(*ρU*_plate_*h*_f_)/(*η*), which is ≈3 for the largest Ω in these experiments. The unidirectional assumption is justified based on the small ratio of radial viscous stress to the azimuthal viscous stress for our experimental conditions:


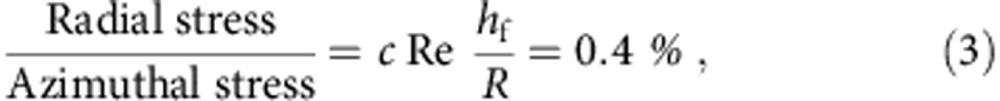


where 
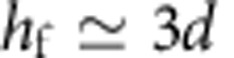
, *R* is the flume radius and 
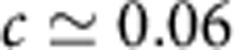
 is an estimated coefficient^5^ that is only weakly dependent on the flow aspect ratio.

### Image analysis

Using a Nikon DSLR 5100 digital camera, we record the real-time positions of single particles by acquiring the fluorescence intensity from a laser dye (concentration ≈1 μM) dispersed in the fluid that is suitable for long data acquisition without significant photobleaching.

To sample fast dynamics near the surface, where the relevant timescale is the settling time of particles over their own diameter *d*/*v*_sed_=0.68 s, we acquire images continuously at 30 Hz for 20 min. To sample slow dynamics in the bed, we acquire single images at a rate of one every 15 s for 10 h or longer.

To detect the positions of the particles with subpixel accuracy, we first correct for effects due to the laser-sheet illumination (see Supplementary Fig. 2), then find particle positions to pixel accuracy by peak-finding above a threshold. The details of the background correction process are shown in Supplementary Fig. 2. It is designed specifically to handle both long-wavelength background intensity variations and intermediate wavelength fluctuations (stripes) due to slight mismatches in the index of refraction of the particles relative to the fluid. On the basis of the raw intensity *M*(*x*,*y*), we define the laser background signal *M*_bk_(*x*,*y*) as the local mean of a rolling disk of diameter 3*d*. In addition, we define the total background intensity fluctuations of the image *σ*_tot_, by performing the usual addition of uncertainties, as a pixel-wise addition of the background and raw image fluctuations: 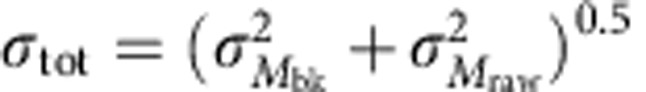. In turn, the background and raw image fluctuations are defined by a local s.d. filter over each image. This filter assigns to every pixel the value of the s.d. of the grey values in a disk-shaped neighbourhood.

We define a scaled fluorescence intensity 
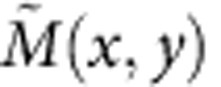
:


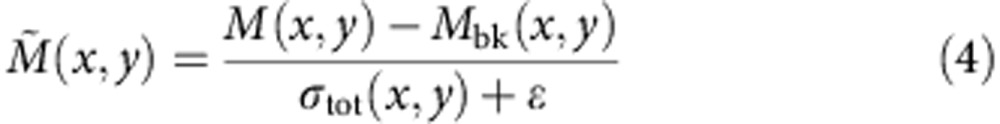


where *ε* is used to avoid numerical division by zero. This normalized image is shown in Supplementary Fig. 3d, where all values above zero are shown as white. Note, its corresponding histogram of grey values shows a clear separation between data above and below background, approximately at zero.

After removing the background, we determine sub-pixel positions using the radial symmetry method^33^. In brief, this method uses the image-gradient vector field to determine the least-squares point of maximal radial symmetry for each particle (see Supplementary Fig. 4). We verify the accuracy of particle detection, by computing the mean-squared-difference *χ*^2^ between the background corrected image and an image generated from the detected particles. If *χ*^2^ is above a threshold, we mask out the particles that we have already found and conduct a further search for smaller, dimmer particles. We do four iterations searching for progressively smaller, dimmer particles, then one last iteration of pixel-accurate detection over the region outside the particles we have already found. A graphical representation of this process is shown in Supplementary Fig. 4a, where the circle colour indicates the iteration number. Most particles are detected on the first iteration, corresponding to in-plane particles, but we find the accuracy of tracks to be improved by searching for additional particles. The relative intensity of particles is sharply peaked below background, and we reject any particle whose relative intensity is above 0.5 relative to the local intensity fluctuations (see Supplementary Fig. 4). We have confirmed that our velocity and concentration profiles are similar when determined using Niblack’s local thresholding algorithm on the raw images, followed by disk detection by convolution with a binary disk-shaped kernel. However, we reduce our overall uncertainty in the velocities and obtain a substantially more accurate estimate of the concentration profile and the position of the surface by using these methods.

We stitch positions at different frames into tracks using Lagrangian particle tracking^34^. This method differs from the Grier–Crocker tracking algorithm^35^ in that it minimizes the mean-squared distance between positions in frames by including an estimate of the velocity in the previous frame. We find this method performs better for data sets such as this, where the range of velocities spans multiple orders of magnitude and no single max-displacement tracking parameter is suitable for all particles.

### Computation of depth-averaged particle flux

Given that we can observe the entire vertical bed structure and dynamics, we choose to apply the rigorous definition of Furbish^36^ to determine the sediment flux, *Q*. As presented in Supplementary Fig. 5a, we define a unique horizontal line at *x*=*x*_p_, where the flux associated with each particle crossing the line is:


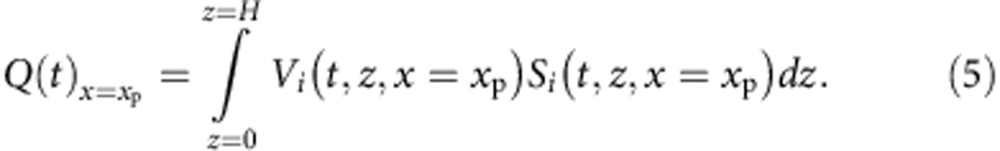


The estimated total flux from the data is the sum of the displacement of *n*_*j*_ particles intersecting the line *x*=*x*_p_ at *t*=*t*_*j*_:


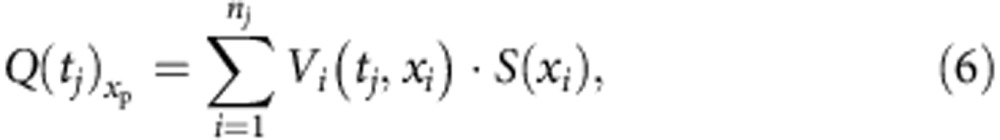


where *S* is the geometrical function defining the cross-sectional area of the individual particle intersecting the line *x*=*x*_p_:


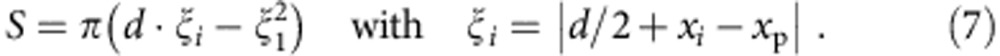


In practice, given the finite acquisition frequency and the wide range and fluctuations of displacements in the system at each given time, three different cases are treated. They are presented in Supplementary Fig. 5b.

The relative error^37^ of the flux is defined as the ratio of the s.e. to the mean value of the flux, *σ*_*Q*_/‹*Q*›, evaluated over the time inverval Δ*t*. This relative error decreases monotonically with increasing Δ*t* for all experiments (see Supplementary Fig. 6). To counter spatial variability, we average nine different positions *x*=*x*_p_ spread over the image frame width to compute the data presented Fig. 2, adding s.e. from the averaging over positions to the respective error bars of each data point. In the main text we use a threshold value of *σ*_*Q*_/‹*Q*›=0.6 to define the convergence timescale of the flux, Δ*t*_conv_; however, the trend for the convergence timescale is not sensitive to choice of threshold.

The fit of the mean particle flux as a function of the Shields number is performed for the function 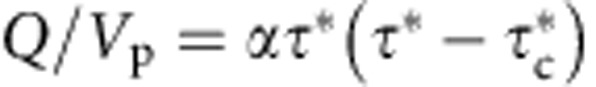, where *V*_p_ is particle volume, we choose 
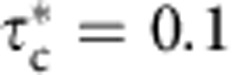
 based on observations from a similar experiment^5^, and we determine the free parameter *α*= 0.91±0.13 s^−1^. The value for *α* is in fair agreement with previous experiments^5^. We do not include the subcritical experiment (τ_*_=0.04) in this fit, as the associated bed-load flux is zero while the flux expression is valid only for positive bed-load flux.

### Detection of the bed surface

The concentration profile *C*(*z*) for a given configuration of particles is determined from a processed binary image, valued at zero outside of particles and one inside of particles. For each elevation *z*, the concentration is determined as the pixel-wise average in the *x* direction. This concentration is the 1D analogue of packing fraction, the fraction of space occupied by the particles.

The surface is defined as the position *z*_s_ at which the concentration crosses fifty percent of its saturated value^38^. As we find the saturated value does not vary significantly from experiment to experiment, we use a fixed threshold of 0.35 to define the surface position. We define *z*_s_ only after averaging the concentration for a time at least as long as Δ*t*_conv_, the flux convergence time. The error bar size is equal to the difference in positions between crossing a concentration equal to 30 and 40%.

### Individual-particle velocity measurement

In a slowly driven granular system, the intermittency of each particle trajectory is important. Figure 1c illustrates the amplitude of the velocity fluctuations for a few particles through time. One can see that there are long periods of immobility, which makes the velocity field measurement delicate. Another difficulty of our experiment is that we capture 2D displacements in a 3D system, which means that particles sometimes move laterally and disappear from the record. To tackle these issues, we measure time steps Δ*T*_*p*,*i*_ of the *i*th displacement of each particle *p* verifying Δ*X*_*p*,*i*_≥3*δx, where *δx* is the spatial resolution limit of our image analysis for the particle centre detection.

We therefore calculate particle velocities as


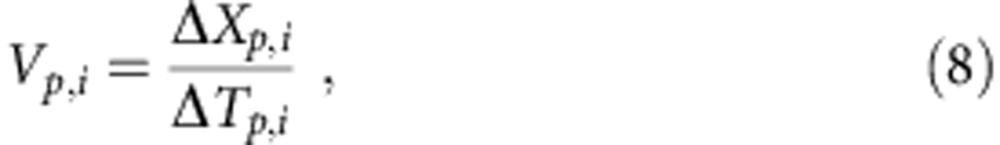


and the associated error


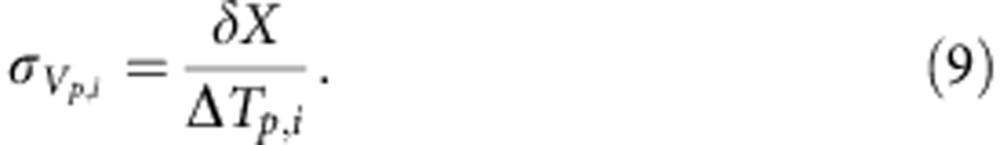


### Ensemble averaging of velocities

At a given depth range *z*_*k*−1_+Δ*z*_*k*_<*z*_*k*_<*z*_*k*+1_−Δ*z*_*k*_, we hypothesize that all the particle displacements detected exhibit a convergent mean velocity. Then we compute the average of the *N*(*z*_*k*_) individual velocities measured during the records in *z*_*k*_±Δ*z*_*k*_


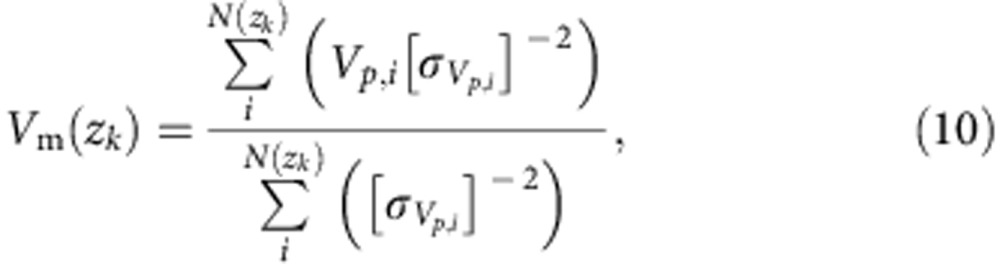


with the associated error


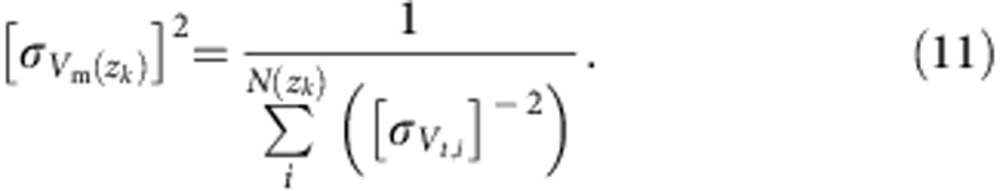


In contrast, some particle displacements are below our detection limit; the total displacement of the tracked particle Δ*X*_*t*_ over the track duration Δ*T*_*t*_ is smaller than our resolution *δx*. To provide a consistent computation of the uncertainties of the measured velocities, we define for each elevation of the profile the minimum velocity we can detect at given elevation *δV*(*z*_*k*_)= *δX*/max(Δ*T*_*t*_(*z*_*k*_)). It corresponds to the fact that the longest track could exhibit a very slow displacement at our detection limit. Practically, it is rather rare to lose the longest tracks; while the slowest motions occur at the flume bottom, we drive the experiment long enough that even these particles typically exhibit a detectable motion. However, a significant number of tracks are too brief for their velocity to be measured. In order not to lose their information, we allocate them the value


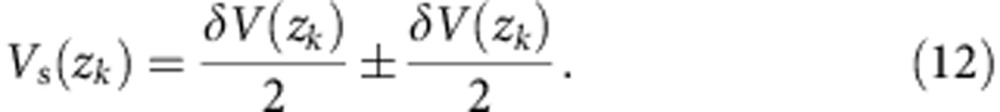


Finally, the velocity field of a given record *n* is computed as an average between these two particles’ populations


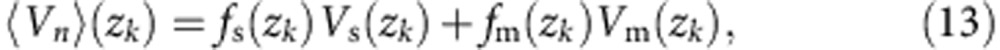


with *f*_s_(*z*_*k*_)=*N*_s_/(*N*_s_+*N*_m_)(*z*_*k*_) the proportion of tracks with an average velocity below our detection limit: Δ*X*_*t*,*j*_/Δ*T*_*t*,*j*_ < *δV*(*z*_*k*_), and conversely, *f*_m_(*z*_*k*_)= *N*_m_/(*N*_s_+*N*_m_)(*z*_*k*_) the proportion of tracks with an average velocity Δ*X*_*t*,*j*_/Δ*T*_*t*,*j*_≥*δV*(*z*_*k*_). (Note that *N*_m_ and *N*_s_ are the number of particles with detected and undetected velocities, respectively.) Accordingly, its associated error is


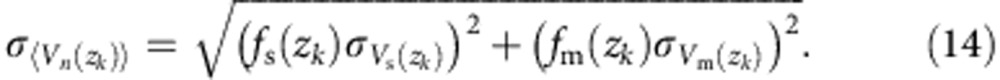


To face the fact that we have a very inhomogeneous data set to analyse, we make an irregular vertical grid (*z*_*k*_,Δ*z*_*k*_) to maintain a fixed number of individual particle velocities *V*_*p*,*i*_ per elevation strip.

Thereafter, we average the two different records using a regular vertical grid (*z*_*l*_,Δ*z*_*l*_):


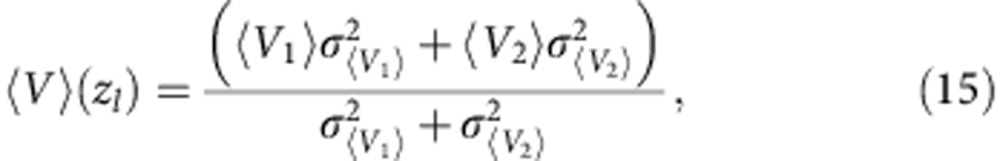


with its associated error:


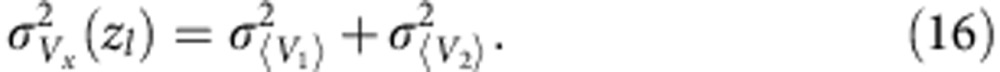


Supplementary Fig. 7a presents all the averaged velocities computed for each individual track, for both records at 30 fps (in red) and 1 image each 15 s (in blue), for the experiment driven at τ*=0.45. We generally capture thousands of individual velocities above our detection limit *δV* for each record. This figure illustrates how the two different records are complementary to probe the entire profile. Superposed black circles on Supplementary Fig. 7b are the final averaged profile ‹*V*›(*z*). In cyan, there are represented the two associated profiles *δV*(*z*) above which we are able to quantify displacement. They naturally increase as the maximum duration of tracks decreases, as particles closer to the surface are more mobile.

### Determination of the critical depth *z*_*c*_

We fit the velocity profiles with the following functional form:


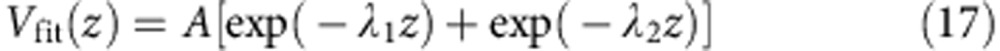


We choose this function because it is able to satisfy a continuous transition between any two exponential decays, including a transition to a uniform value. Restricting *λ*_1_ and *λ*_2_ to positive values guarantees that the function itself is continuous and monotonically decreasing, and its derivative (the shear-rate) is also continuous and monotonically decreasing. We fit the data using this function by solving the nonlinear least-squares minimization of *χ*^2^ using the Nelder–Mead simplex method. We define *χ*^2^ using equal weights in log-space, by fitting *y*_data_=log(*V*_data_) against *y*_fit_=log(*V*_fit_)=log(exp(−*λ*_1_*z*)+exp(−*λ*_2_*z*))+*A*, with three free parameters *λ*_1_, *λ*_2_ and *A*. The *χ*^2^ for the fits is computed over the non-uniform grid of *z*-values by using Simpson’s method of integration.

The critical depth *z*_c_ is defined as the point of maximum curvature in log-space:


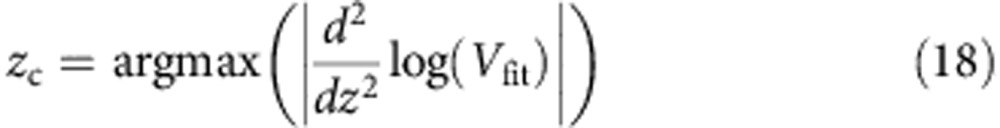


### Determination of the critical viscous number *I*_*c*_

We estimate *I*_c_ from the average of the values observed nearest to *z*_c_, from the intersection of the regression lines for each profile at different Shields numbers, and from the intersection of the double exponential fits. Within their uncertainties, the three methods converge on the same value of *I*_c_ as 1 × 10^−7^.

### Comparison of creeping flux relative with bed-load flux

To quantify the net importance of the creeping regime relative to the bed-load regime, we compute the flux of both by integrating our concentration and velocity profiles. Therefore, we write the creeping flux as:


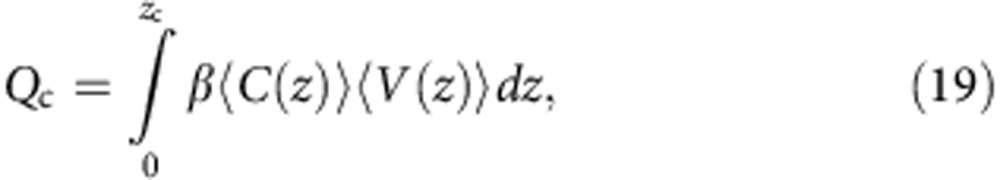


and the bed-load flux:


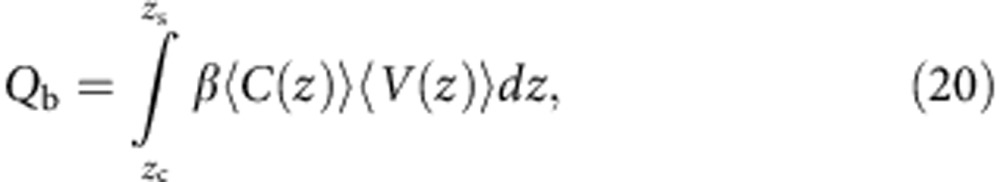


with *β* a coefficient close to 1, introduced above.

The flux profiles are represented Supplementary Fig. 8a. Supplementary Figure 8b presents both fluxes, creeping and bed load, as a function of the Shields number. We can see that while the bed-load flux increases notably with the Shields number, the creeping flux remains constant. As a consequence, the proportion of the creeping flux *Q*_c_ relative to the sum *Q*_c_+*Q*_b_, represented in Supplementary Fig. 8c, increases rapidly as the Shields number decreases towards the threshold of bed-load transport. For the experiment at *τ*=0.04, the proportion of creeping reaches 100%.

## Author contributions

All authors contributed to experimental design and writing the manuscript. M.H. performed the experiments. M.H. and C.P.O. analysed and interpreted data. D.J.D. and D.J.J. supervised the research and contributed to data interpretation. D.J.J. managed the project.

## Additional information

**How to cite this article:** Houssais, M. *et al.* Onset of sediment transport is a continuous transition driven by fluid shear and granular creep. *Nat. Commun.* 6:6527 doi: 10.1038/ncomms7527 (2015).

## Supplementary Material

Supplementary Figures Supplementary Figures 1-8

Supplementary Movie 1 Time-lapse video of 1.5 mm PMMA particles under shear in an index-matched, but not density-matched medium. The shear stress is tau*=0.44. The real duration of the video is 22 hours, but its playback time is 30 seconds.

Supplementary Movie 2 Real-time video of 1.5 mm PMMA particles under shear in an index-matched, but not density-matched medium. The shear stress is tau*=0.44. The real duration of the video is 20 seconds, the same as its playback time.

Supplementary Movie 3 Time-lapse video of 1.5 mm PMMA particles under shear in an index-matched, but not density-matched medium. The shear stress is tau*=0.04. The real duration of the video is 205 hours, but its playback time is 30 seconds.
