# Supplementary material for: Onset of sediment transport is a continuous transition driven by fluid shear and granular creep
Source: Nat Commun. 2015 Mar 9;6:6527. doi: 10.1038/ncomms7527 (PMC4366508; doi:10.1038/ncomms7527)
Supplement: Supplementary Figures — 1-8 [file ncomms7527-s1.pdf]

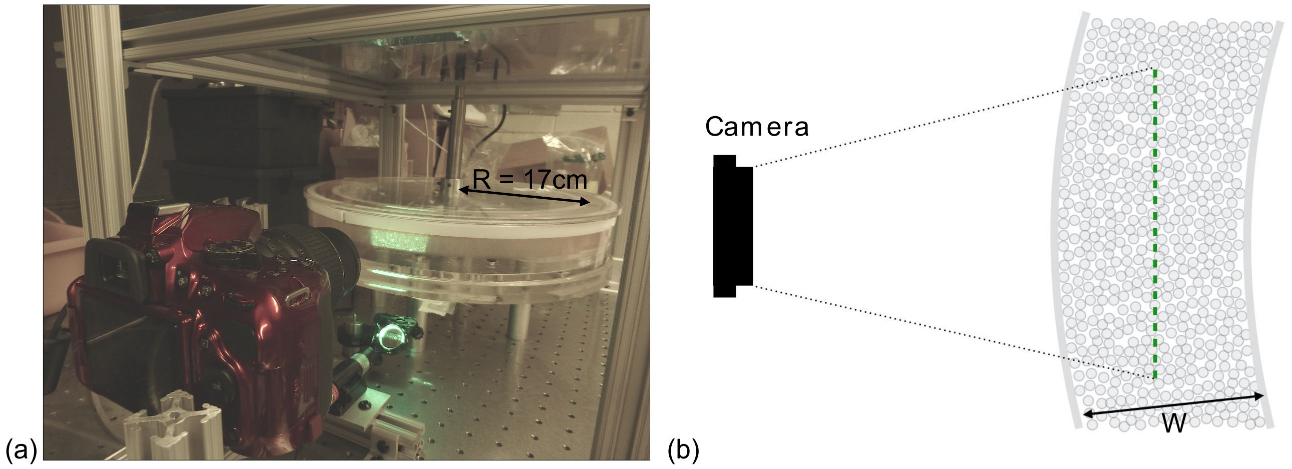

Supplementary Figure 1: a) Image of the annular flume illuminated by a green laser sheet. b) Top-view scale sketch of the acquisition setup. The laser sheet passes through the midplane of the channel.

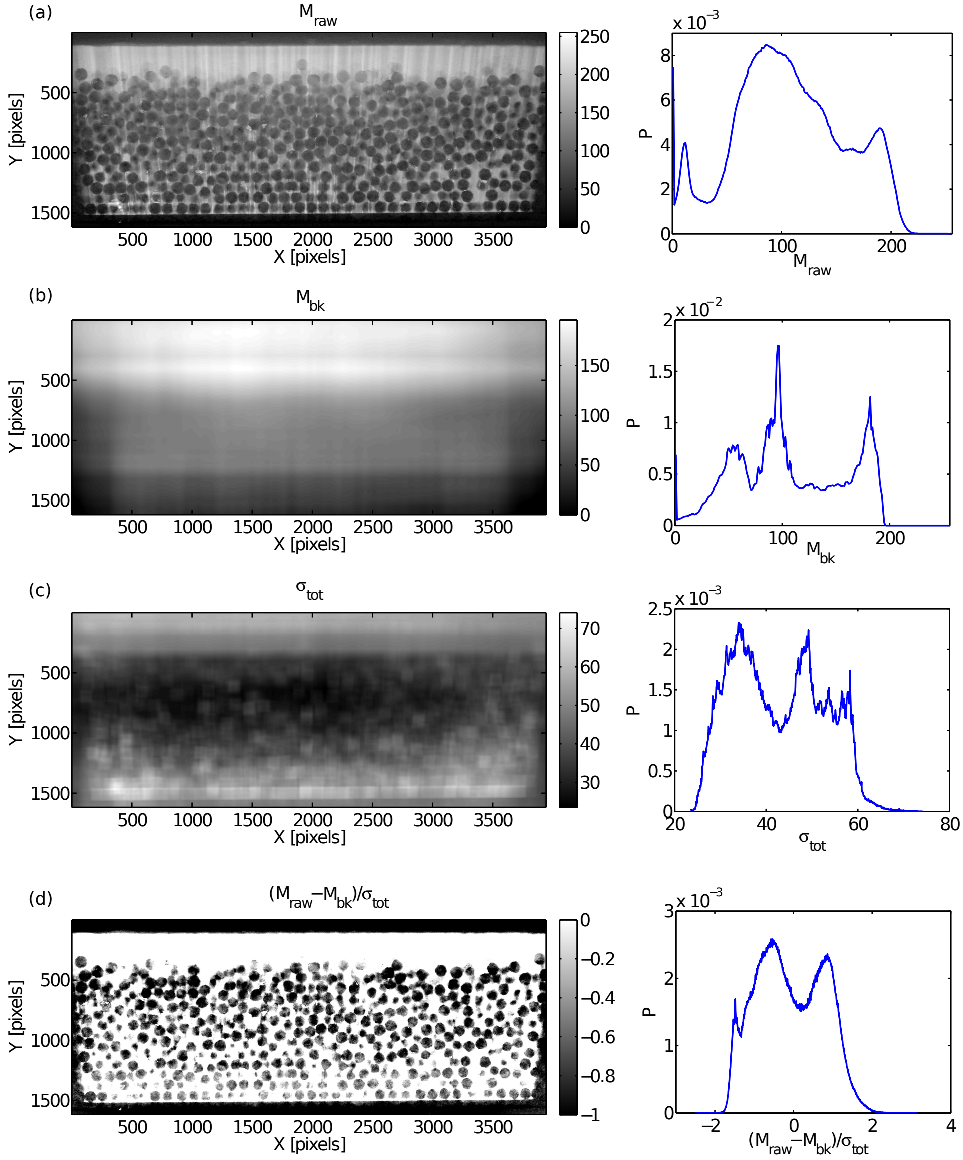

Supplementary Figure 2: Normalization of raw images. a) Raw experimental image. b)Estimated background image from raw image. c) Estimated σ_{tot} from the local standard deviation of the raw image and the background image. d) Normalized relative fluorescence intensity, displayed in the range from -1 to 0, such that all values greater than zero (background fluorescence) show as solid white. The right-column shows the histogram of the grey values in the corresponding left-column image.

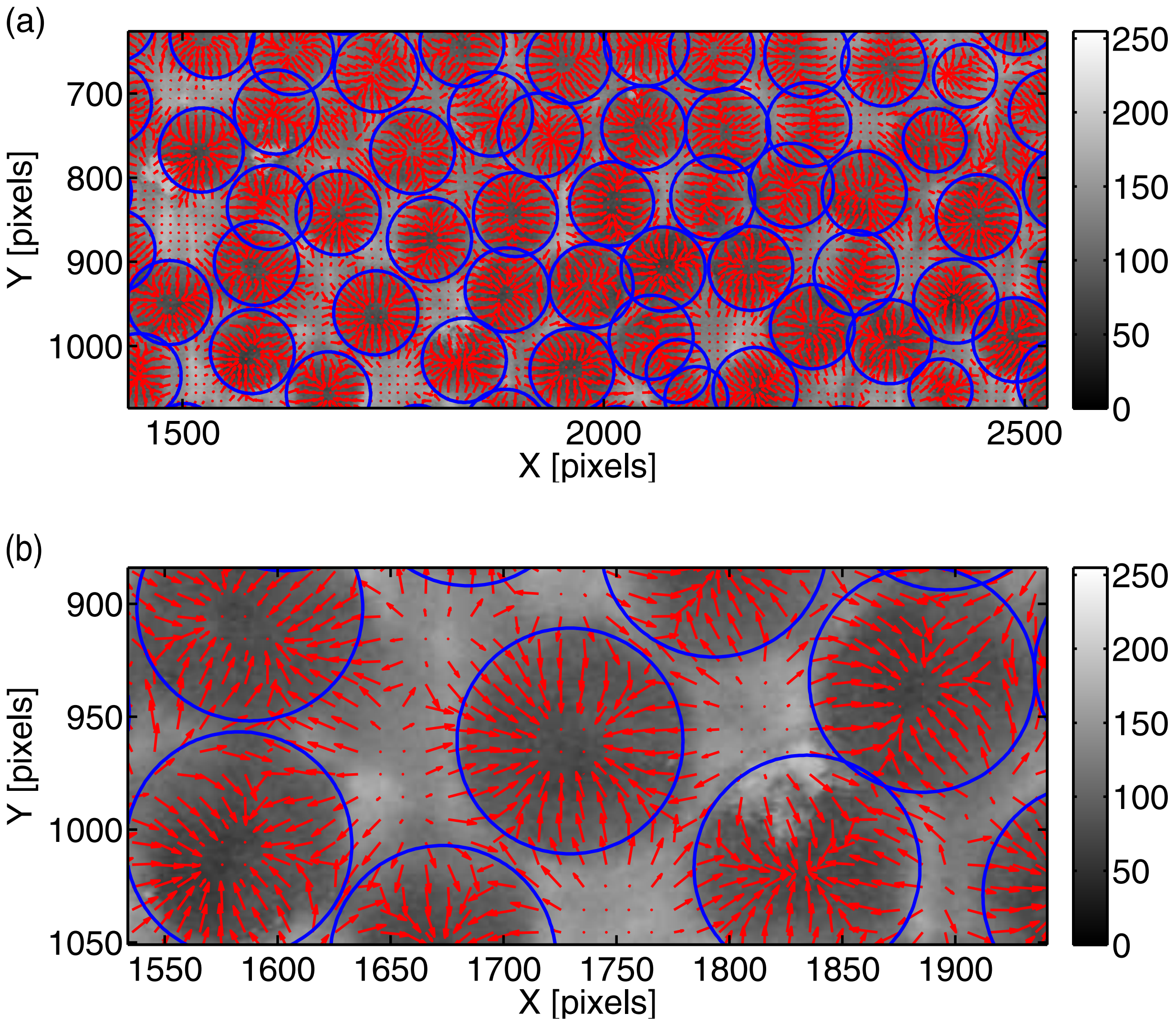

Supplementary Figure 3: Vector field of intensity gradient as arrows, and detected particles as circles overlayed on a raw image for (a) a region $\approx 5d \times 10d$ and (b) a region $\approx 1.5d \times 5d$, both near the centre of the pack.

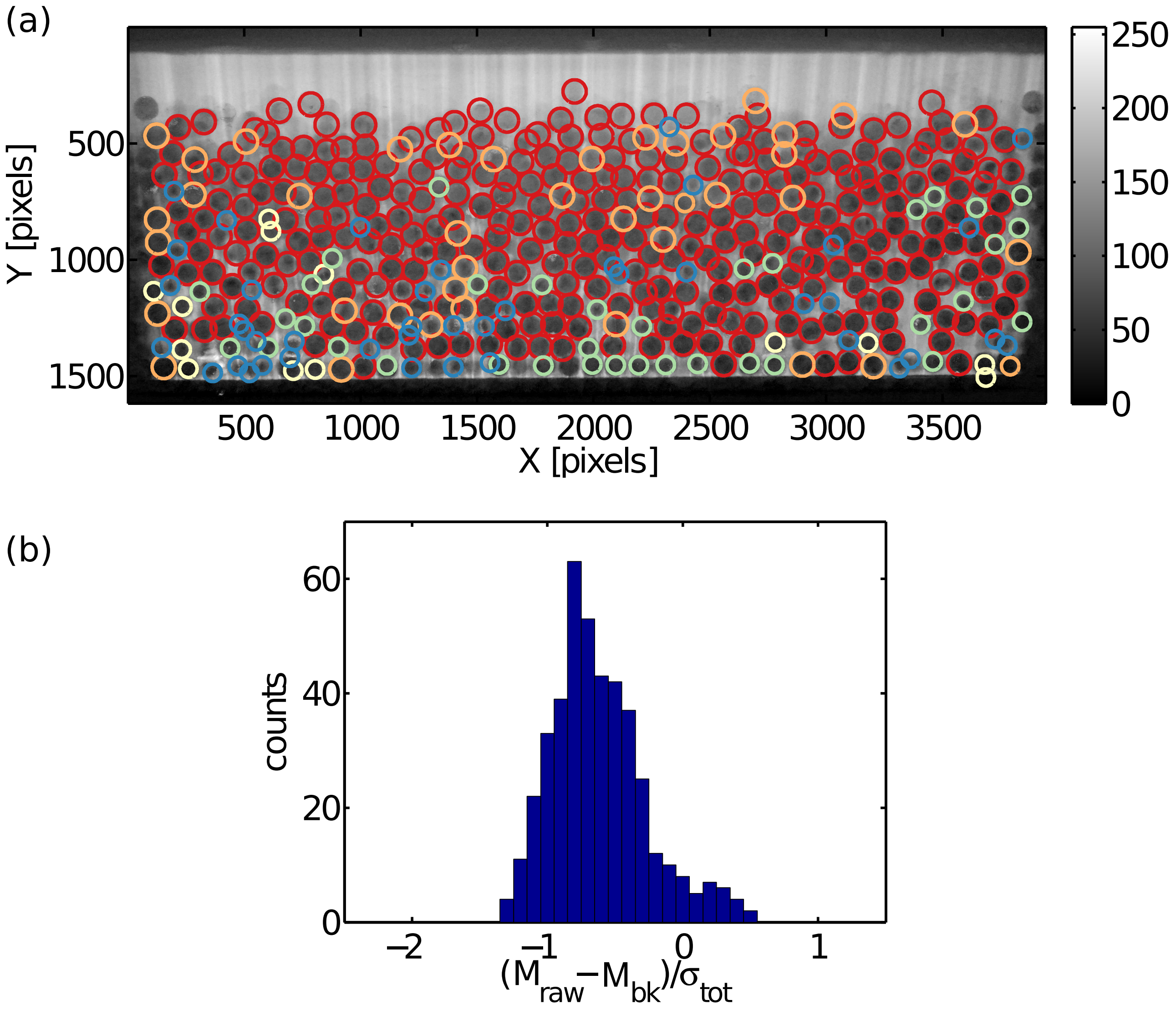

Supplementary Figure 4: Final particle positions detected from our methods. (a) Detected particles plotted as circles overlayed on a raw image. The iteration number at which particles are detected is indicated by the colour (grey level) of the circle, which varies from red (dark grey) circles for the first iteration to blue (light grey) circles for the fifth iteration. (b) Histogram of average intensity inside the detected particles relative to the local background intensity I_{bk} and local intensity fluctuation scale σ_{tot}.

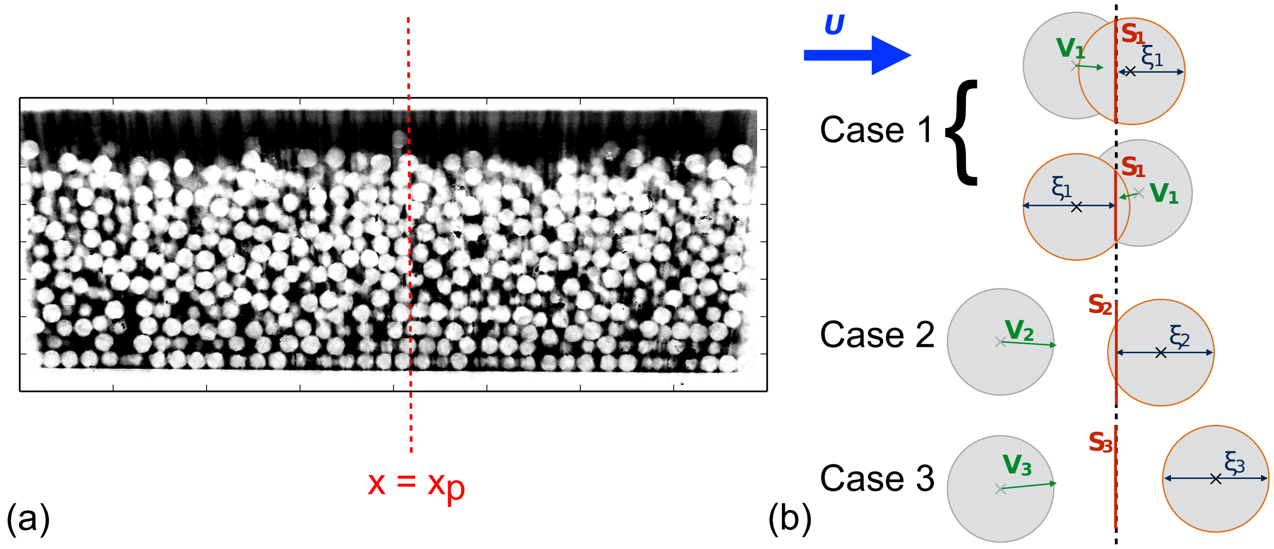

Supplementary Figure 5: Flux time series measurement. a) Definition of a local line $x = x_p$ in the image series. b) In practice, particles that cross the line between $t_j - \Delta t$ and t_j can do so in different configurations. Case 1: they intersect the line at both times, and can present a positive or negative displacement. Case 2: They intersect the line only at $t = t_j$. In both these cases, the function S_i is arbitrarily computed from the particle position at $t = t_j$. Case 3 : between $t_j - \Delta t$ and t_j the particle entirely crosses the line, in that case the maximum of S_i $(\pi \cdot d^2/4)$ is used.

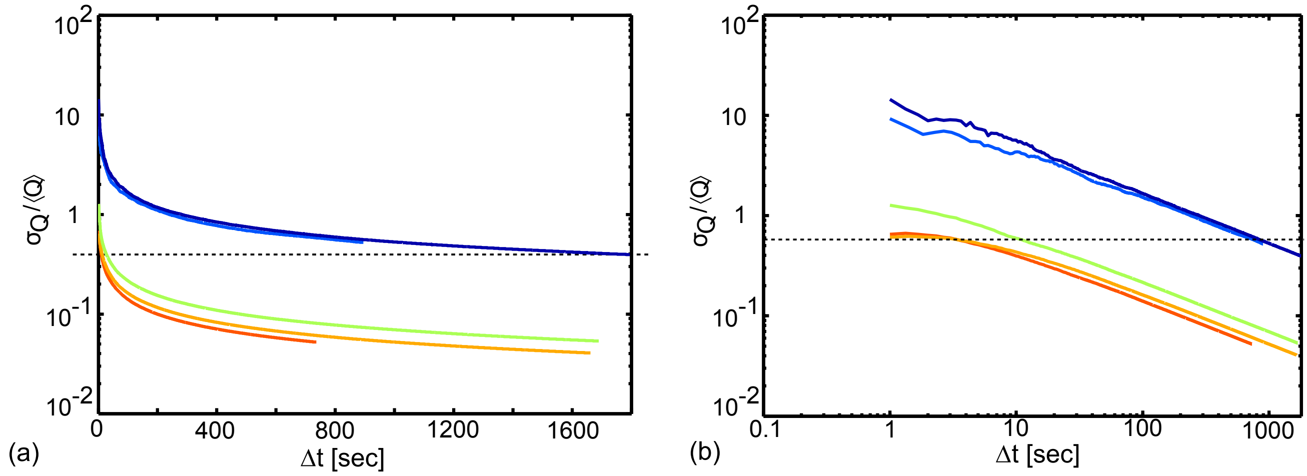

Supplementary Figure 6: Convergence time measurement. a) Ratio $\sigma_Q/\langle Q\rangle$, for a given position x_p as a function of the time window used for the averaging, based on a Monte Carlo sampling of the data. Colours indicate the different experiments as referred to in Fig. **??**. b) The same figure is represented with a logarithmic time scale. The dashed line represents the threshold (0.6) used to identify the convergence time. To compute the Δt_{conv} values reported in Fig. **??**b, we average the values obtained at 9 different positions x_p.

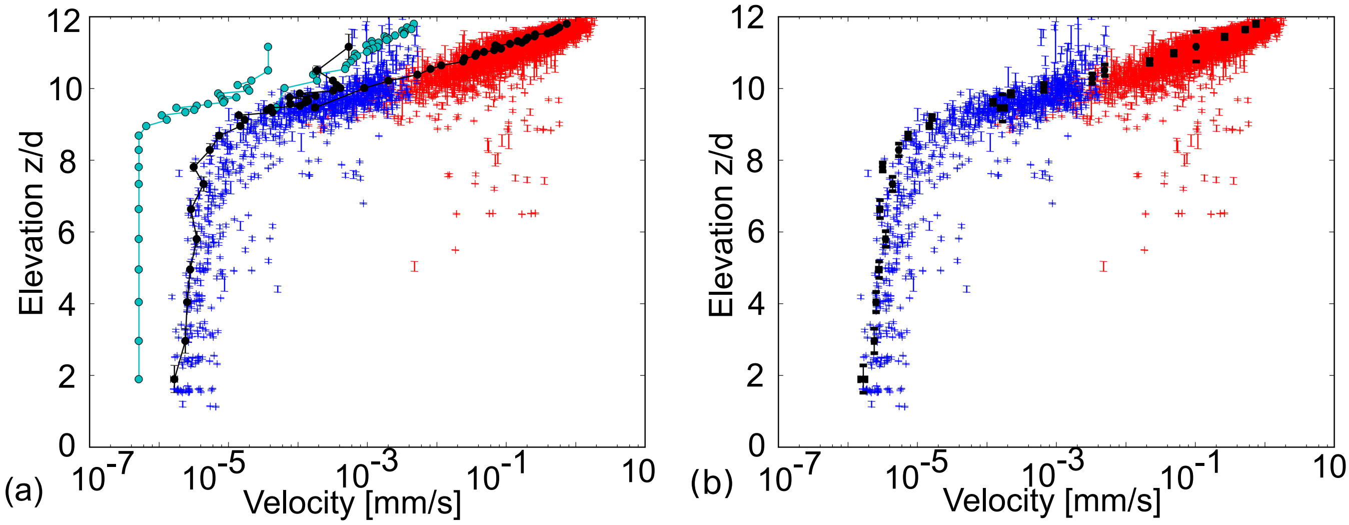

Supplementary Figure 7: Computation of the averaged velocity profile. a) Averaged velocities computed for each individual track at 30 fps (red) and 1 image each 15 seconds (blue), for the experiment driven at $\tau^* = 0.44$. Black circles are the average profiles per record $\langle V_i \rangle$, $i = 1, 2$. In cyan are represented the two profiles of limiting resolution velocities $\langle \delta V_i \rangle$, $i = 1, 2$. Measured velocities are clearly above the limiting resolution. b) The final averaged velocity profile $\langle V \rangle$.

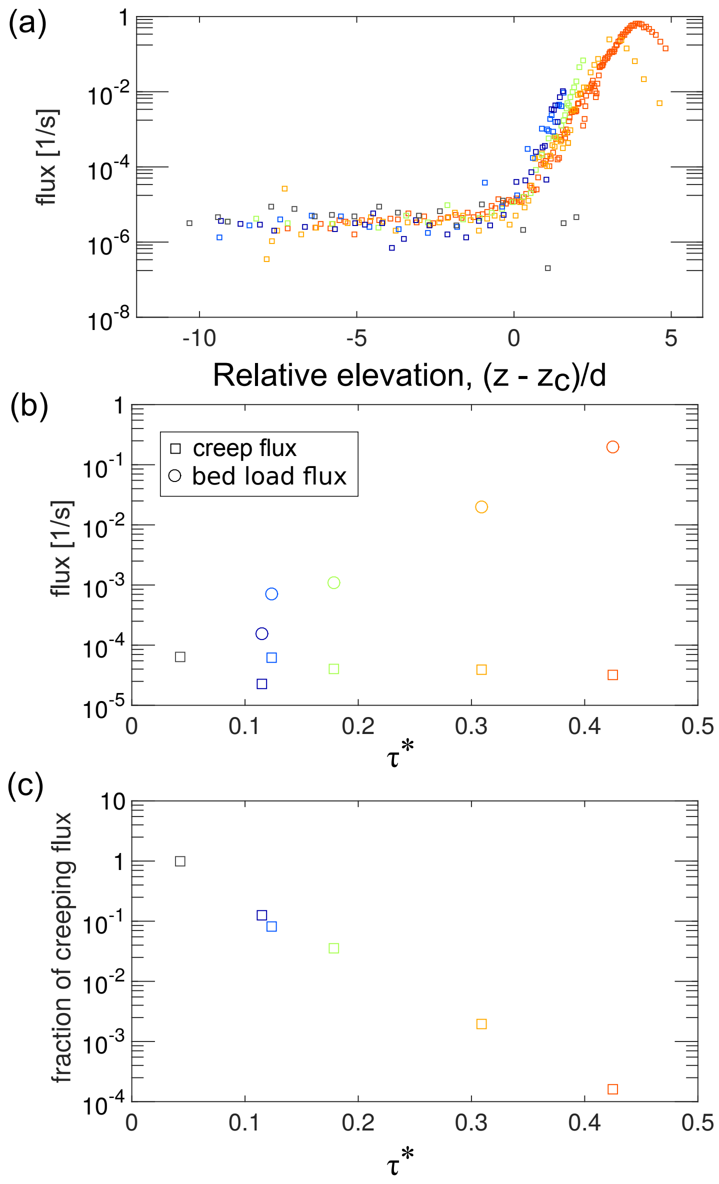

Supplementary Figure 8: . Depth-averaged sediment flux in different regimes. a) Ensemble-averaged sediment flux $q(z) = C(z)v(z)/d$ as a function of elevation shifted by z_c. For the sub-threshold experiment, we use $z_c = z_s$. The Shields number is indicated by colour. b) Flux integrated over an entire phase as a function of Shields number. Squares indicate creeping flux. Circles indicate bedload flux. c) Fraction of creeping flux to total flux as a function of Shields number.
